# A study on the academic innovation ability and influencing factors of public health graduate students based on nomograms: a cross-sectional survey from Shandong, China

**DOI:** 10.3389/fpubh.2024.1429939

**Published:** 2024-08-23

**Authors:** Xinyu Wang, Pengxin Geng, Xingyue Chen, Weiqin Cai, Hongqing An

**Affiliations:** ^1^School of Public Health, Shandong Second Medical University, Weifang, China; ^2^Institute of Public Health Crisis Management, Shandong Second Medical University, Weifang, China; ^3^School of Management, Shandong Second Medical University, Weifang, China

**Keywords:** nomogram, academic innovation ability, graduate student, public health, medical education reform, cross-sectional study, China

## Abstract

**Background:**

In recent years, the impact of the COVID-19 pandemic and various public crises has highlighted the importance of cultivating high-quality public health talents, especially those with innovative capabilities. This study focuses on the academic innovation ability of public health postgraduate students, which can provide important theoretical support for the cultivation of more public health workers with high innovative capabilities.

**Methods:**

From May to October 2022, a cluster sampling method was used to select 1,076 public health postgraduate students from five universities in Shandong Province. A self-designed questionnaire survey was conducted. A chi-square test and binary logistic regression analysis were used to analyze the influencing factors of students’ academic innovation ability. Based on these factors, a nomogram was constructed to intuitively demonstrate the impact of these complex factors on students’ innovation ability.

**Results:**

The results showed that gender, whether serving as a student leader, teacher-student relationship, academic motivation, learning style, academic environment, and teaching mode were the influencing factors of postgraduate students’ academic innovation ability. The column-line diagram (AUC = 0.892, 95% CI = 0.803 ~ 0.833) constructed based on the above influencing factors has good differentiation. The area under the ROC curve is 0.892 (95% CI = 0.803 ~ 0.833), and the calibration curve shows that the predicted value is the same as the measured value.

**Conclusion:**

The nomogram constructed in this study can be used to predict the academic innovation level of public health graduate students, which is helpful for university education administrators to evaluate students’ academic innovation ability based on nomogram scores and carry out accurate and efficient training.

## 1 Introduction

The COVID-19 pandemic has made countries around the world realize that having a well-rounded public health workforce is an important guarantee for responding to public health crises, especially those with high-quality public health talents who can lead innovation and drive technological progress (1). As high-level talents in the future field of public health, graduate students in public health cultivate their academic innovation ability during their time at university so that they can become more innovative public health workers to respond to various public health disasters (2).

Over the past decade, the scale of postgraduate education in China has increased rapidly, making it the second-largest country in the world in this area of education (3). Unfortunately, the overall quality of graduate education in China is inadequate, and graduate students’ lack of innovation ability is considered a significant problem in graduate education (4). There is an urgent need to address the obvious contradiction between the lack of public health human innovation capacity and the increasing complexity of local public health emergency tasks (5, 6). Therefore, in-depth research must be conducted on the cultivation of innovation ability in graduate students’ education in public health, and the problems and influencing factors in students’ training must be clarified.

The existing research on the influencing factors of graduate students’ innovation ability mainly focuses on the influence of mentor support and guidance style, creative self-efficacy, cognitive skills, and early research experience on graduate students’ innovation ability (7–9). The psychologist Amabile proposed the componential theory of creativity (10) and Woodman proposed the interactional model of creativity (11), revealing the influence mechanism of personal endowment and external environmental variables on innovation ability, including knowledge, intrinsic motivation, cognitive ability, and personality. The external environment includes external evaluation, reward, and supervision. Subsequent studies have also explored the influencing factors affecting the academic innovation ability of public health graduate students from both individual and environmental aspects. A systematic review of this literature shows that individual factors mainly include academic motivation, learning style, and scientific research participation (12, 13), while environmental factors mainly include academic environment, teaching mode, teacher-student relationship, academic support from tutors, academic platforms, training models, and degree policy requirements (14–17).

It is worth noting that although a large number of studies have explored the influencing factors of graduate students’ innovation ability, lack of research in the field of public health persists. Most of the existing studies do not distinguish between disciplines but include all graduate students in various disciplines in the research scope to explore the cultivation of such students’ innovation ability and analyze the influencing factors. In addition, many studies tend to explore the impact on innovation ability from only one perspective, such as external factors (e.g., mentoring style) or individual factors (e.g., self-efficacy) (18–20). Research on graduate students’ innovation ability in the field of public health from the perspective of comprehensive factors is lacking.

Nomograms can quantify the risk of an event through a variety of predictors, visually showing the results of logistic regression (21). At present, nomograms are widely used in the prediction of clinical diseases and show good predictive ability in the study of non-clinical problems, such as the prediction of undergraduate students’ self-regulated learning level (22), the risk of adolescent bullying (23), and primary and secondary school students’ suicidal tendency (24). Therefore, nomograms can be applied to the quantitative prediction and evaluation of the influencing factors of academic innovation to provide more accurate theoretical support for the improvement of the innovation level of public health graduate students.

## 2 Materials and methods

### 2.1 Research design and data sources

From May to October 2022, this cross-sectional study used a cluster sampling method to select five universities in Shandong Province with this the public health major as survey sites; all public health graduate students from the five universities were selected as survey participants. In light of strict COVID-19 control measures, data were collected online through the use of Wenjuanxing. Wenjuanxing is a professional online survey tool with comprehensive data collection and privacy protection features. The purpose of this study and the privacy policy were explained to participants, who could decide whether or not to complete the questionnaire after reading the questionnaire instructions. Before the start of the questionnaire, participants were required to check the following: “I have read and agree to participate in this study,” indicating that they were willing to participate and provide informed consent. To ensure the quality of the questionnaire, questionnaires with a filling time of less than 5 min and incomplete filling were excluded.

We used WeChat to distribute the surveys, given the high popularity and convenience of the platform. First, we established close cooperation with the five universities in Shandong Province with public health-related majors and appointed investigators from each university as our liaison. These investigators were rigorously screened and trained to ensure that they had a thorough understanding of the purpose of the study, the content of the questionnaire, and the distribution process. After, the investigators contacted the heads of public health disciplines at their respective institutions to introduce the importance of the study, the content of the questionnaire, and the precautions for filling it out. After, the heads of the disciplines forwarded the link to all class representatives in each school year to ensure that every student would promptly receive the link to the questionnaire and clearly understand the meaning and requirements of filling it out. From a total of 1,255 graduate students, 1,150 questionnaires were collected, with a response rate of 91.64%. Seventy-four invalid questionnaires were eliminated; 1,076 valid questionnaires were valid, with an effective rate of 93.57%.

### 2.2 Questionnaire

Combined with the characteristics of expert consultation, preliminary research foundation (25–29), and the subjectivity, initiative, and practicality of Chinese public health graduate students, we compiled the Questionnaire on Academic Innovation Ability of Public Health Graduate Students. After the questionnaire was completed, we conducted a pilot test at Shandong Second Medical University. In response to the problems in the pilot test process, we modified the questionnaire. The questionnaire consisted of three parts: demographic information, questionnaire on academic innovation ability, and questionnaire on influencing factors of academic innovation ability.

The questionnaire on academic innovation ability included four dimensions: personal traits (4 items), professionalism (6 items), basic abilities (5 items), and thinking characteristics (2 items). Each item adopted a 5-level Likert scoring system, and was assigned 1–5 points ranging: “very disagreeable,” “somewhat dissatisfied,” “somewhat compliant,” “compliant,” and “very consistent.” The higher the score, the higher the individual academic innovation ability. The total score ranged from 43 to 215, and a score of >129 indicates high academic innovation ability; otherwise, scores were recorded as low academic innovation ability (30, 31). In this study, Cronbach’s alpha of the four dimensions of personal traits, professional qualities, basic abilities, and thinking characteristics were 0.967, 0.960, 0.951, and 0.901, respectively, indicating that the questionnaire had good reliability. The Kaiser-Meyer-Olkin (KMO) value was 0.984, which verified the construct validity of the questionnaire construction.

The design of the influencing factors of academic innovation ability was mainly based on the previously published literature represented by Amabile’s innovation ability component theory and Woodman’s innovation ability interaction model. The influencing factors were divided into two dimensions: individual and environment. Among them, the individual dimension mainly covers sex, degree, degree type, student leadership, scientific research participation, academic motivation, and learning style. The environmental dimension mainly covered academic platform, teacher-student relationship, tutor academic support, academic environment, training mode, degree requirement policy, and teaching mode. The above factors formed the academic innovation ability questionnaire, and the content of the survey was mainly in the form of students’ own attitudes and cognitions. In this study, Cronbach’s alpha of the questionnaire was 0.847, indicating that the scale has good internal consistency and reliability. The KMO value was 0.831, which verified that the questionnaire had good construct validity.

### 2.3 Statistical methods

SPSS26.0 and R version 4.3.1 software were used for data analysis; the measurement data were expressed as mean ± standard deviation and the count data were expressed as the number of cases and percentage (%). The *t*-test was used for the comparison of quantitative data between groups; The *χ*^2^ test was used for the comparison of categorical data between groups, and binary logistic regression was used for the analysis of influencing factors; the difference was statistically significant, with *p* < 0.05. According to the identified influencing factors, a nomogram was constructed to predict the academic innovation ability of graduate students in public health. The receiver operating characteristic (ROC) curve, area under the ROC curve (AUC), and calibration curve were used to evaluate the prediction accuracy and consistency of the model.

### 2.4 Ethics approval

This study was approved by the Ethics Committee of Weifang Medical University (No. 2021YX130, Weifang Medical University was renamed Shandong Second Medical University in December 2023). Prior to the study, all participants were informed of the study’s research purpose and content and provided informed consent online.

## 3 Results

### 3.1 Statistical analysis of the description of the academic innovation ability of graduate students in public health

As shown in Table 1, the graduate students’ academic innovation ability scored 3.50 ± 0.71. Among the four dimensions, professionalism scored the highest, with an average score of 3.65 ± 0.73, while the other dimensions scored an average of 3.5 or less; personal traits scored an average of 3.31 ± 0.86, basic abilities averaged 3.47 ± 0.76, and thinking characteristics scored an average of 3.48 ± 0.81. Among the secondary indicators, postgraduate career planning scored the highest, with a mean score of 3.82 ± 0.87. Psychological adaptation and decision-making ability scored the lowest, with 3.29 ± 0.89 and 3.29 ± 0.96, respectively.

**Table 1:** Score on the academic innovation ability of public health graduate students.

First-level indicators	Secondary indicators	Low academic innovation M ± SD	High academic innovation M ± SD	All M ± SD
Personal traits	Self-control	2.88 ± 0.64	4.10 ± 0.63	3.31 ± 0.86
	Psychological adjustment	2.83 ± 0.64	4.13 ± 0.61	3.29 ± 0.89
	Trait of flexibility	2.94 ± 0.61	4.16 ± 0.58	3.38 ± 0.84
	Achievement oriented	3.06 ± 0.59	4.29 ± 0.45	3.50 ± 0.80
Professionalism	Foresight of science	3.17 ± 0.64	4.29 ± 0.46	3.57 ± 0.79
	Ethics of work	3.30 ± 0.76	4.33 ± 0.49	3.67 ± 0.84
	Awareness of service	3.23 ± 0.69	4.35 ± 0.48	3.63 ± 0.82
	Professional responsibility	3.00 ± 0.67	4.26 ± 0.53	3.45 ± 0.87
	Career planning	3.51 ± 0.88	4.37 ± 0.51	3.82 ± 0.87
	Working in a team	3.42 ± 0.75	4.36 ± 0.48	3.76 ± 0.80
Basic abilities	Ability to execute	3.30 ± 0.66	4.34 ± 0.47	3.67 ± 0.78
	Communication skills	3.28 ± 0.68	4.29 ± 0.51	3.64 ± 0.79
	Ability to learn	2.96 ± 0.69	4.13 ± 0.63	3.37 ± 0.87
	Decision-making skills	2.86 ± 0.78	4.06 ± 0.76	3.29 ± 0.96
	Outreach capability	2.95 ± 0.77	4.12 ± 0.69	3.37 ± 0.93
Thinking characteristics	Critical creative thinking	3.03 ± 0.67	4.21 ± 0.54	3.45 ± 0.84
	Information integration management	3.11 ± 0.76	4.22 ± 0.57	3.50 ± 0.88

**Figure 1:**
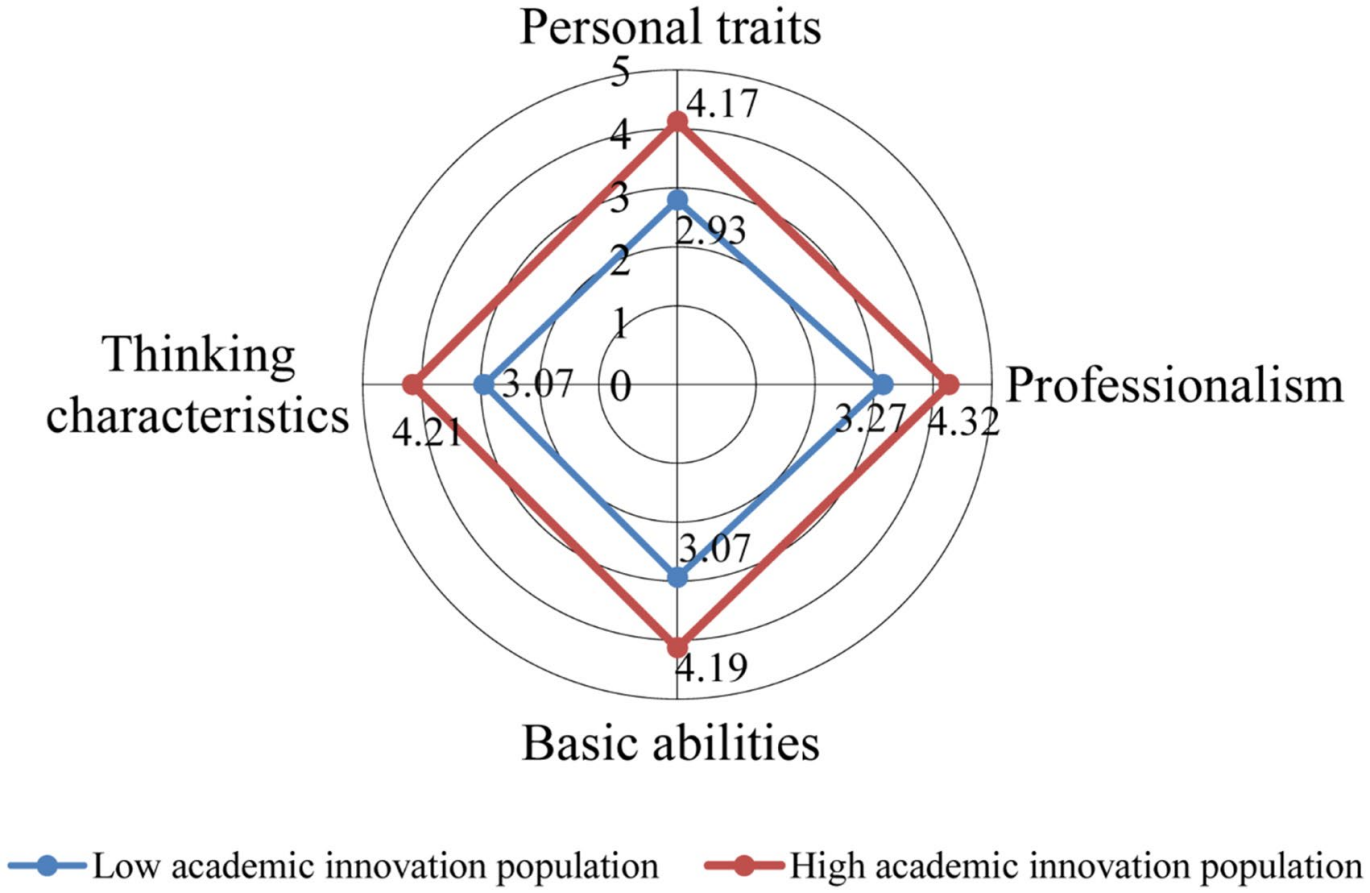
Radar chart of the scores of the two types of graduate students on academic innovation ability.

The radar chart in Figure 1 shows the academic innovativeness scores of different postgraduate students. As shown in Figure 1, there is a difference in the academic innovativeness scores of different students. Students with low academic innovativeness scored between 2 and 3, while all the students with high academic innovativeness scored more than 4. The average graduate student score was about 3. Across all items, both categories of postgraduate students had higher mean scores on professionalism (3.27 and 4.32, respectively) and lower scores on personal traits, which were particularly pronounced for postgraduate students with low levels of academic innovativeness.

As shown in Table 2, a total of 1,076 postgraduate students were surveyed in this study, including 753 cases in the modeling group and 323 cases in the validation group. There were 409 cases (38%) of males and 667 cases (62%) of females. Most of the study participants were in the master’s degree segment, accounting for 92.2%. Professional degrees had the highest number, accounting for 73%. More graduate students did not serve as student leaders, accounting for 60.7%. Among the postgraduate students with different genders, degree, whether they served as student cadres, academic platforms, teacher-student relationships, scientific research participation, academic motivation, learning style, academic support from supervisors, academic environment, training mode, degree requirement policy perception, and teaching mode were statistically significant (*p* < 0.05) in different academic innovation capabilities.

**Table 2:** General characteristics and univariate analysis of public health graduate students.

Categories	Low academic innovation ability (*N* = 693)	High academic innovation ability (*N* = 383)	*p*-value
Gender*			
Men	231 (33.33)	178 (46.48)	<0.001
Women	462 (66.67)	205 (53.52)	
Degree*			
Master	638 (92.06)	354 (92.43)	0.831
Doctor	55 (7.94)	29 (7.57)	
Degree type*			
Academic degree	204 (29.44)	86 (22.45)	0.014
Professional degree	489 (70.56)	297 (77.55)	
Whether serving as a student leader*			
Yes	238 (34.34)	185 (48.30)	<0.001
No	455 (65.66)	198 (51.70)	
Academic platform**	3.39 ± 0.82	3.73 ± 0.92	<0.001
Teacher-student relationship**	3.38 ± 0.82	3.79 ± 0.88	<0.001
Scientific research participation**	3.79 ± 0.76	3.93 ± 0.64	<0.001
Academic motivation**	3.17 ± 0.78	4.04 ± 0.75	<0.001
Learning style**	3.20 ± 0.77	4.09 ± 0.67	<0.001
academic support from supervisors**	3.11 ± 0.81	3.91 ± 0.81	<0.001
Academic environment**	2.97 ± 0.75	3.80 ± 0.89	<0.001
Training model**	3.00 ± 0.77	3.79 ± 0.91	<0.001
Degree requirement policy**	3.06 ± 0.81	3.81 ± 0.87	<0.001
Teaching model**	3.04 ± 0.82	3.77 ± 0.82	<0.001

The supplementary footnotes are as follows: *describe the characteristics of participants with n(%); **describe the characteristics of participants with M±SD.

### 3.2 Analysis of factors influencing public health postgraduate students’ academic innovation capacity

The results of the logistic regression analyses in Table 3 show that gender, whether or not they are student leaders, teacher-student relationships, academic motivation, learning styles, academic environment, and teaching modes are influential factors in the academic innovativeness of public health postgraduate students. Males were 1.769 times more likely than females to have high levels of innovation (95% CI = 1.255–2.492). Graduate students who served as student leaders were 1.616 times more likely to have high levels of academic innovativeness than those who did not (95% CI = 1.149, 2.273). For every 1-point increase in the self-scoring score of the student-faculty relationship, the likelihood of having a high level of academic innovativeness increased by 1.83 times (95% CI: 1.478–2.264). In addition, the likelihood of having a high level of academic innovativeness increases by 2.04 times (95% CI: 1.567–2.657), 2.909 times (95% CI: 2.185–3.872), and 1.646 times (95% CI: 1.288–2.103) for each 1-point increase in the self-rating scores of Academic Motivation, Learning Styles, Academic Environment, and Teaching Mode, respectively, 2.331 times (95% CI: 1.849–2.939).

**Table 3:** Logistic regression analysis of the influencing factors of academic innovation ability of graduate students.

Categories	*β*	SE	Wald	*p*	OR (95%CI)
Sex (Women)	0.570	0.175	10.630	0.001	1.769 (1.255, 2.492)
Whether serving as a student leader (No)	0.480	0.174	7.614	0.006	1.616 (1.149, 2.273)
Teacher-student relationships	0.604	0.109	30.840	<0.001	1.830 (1.478, 2.264)
Academic motivation	0.713	0.135	28.060	<0.001	2.040 (1.567, 2.657)
Learning style	1.068	0.146	53.511	<0.001	2.909 (2.185, 3.872)
Academic environment	0.498	0.125	15.868	<0.001	1.646 (1.288, 2.103)
Teaching model	0.846	0.118	51.230	<0.001	2.331 (1.849, 2.939)
Constant quantity	−14.174	0.875	262.219	<0.001	

### 3.3 Construction of nomogram of academic innovation ability of graduate students in public health

**Figure 2:**
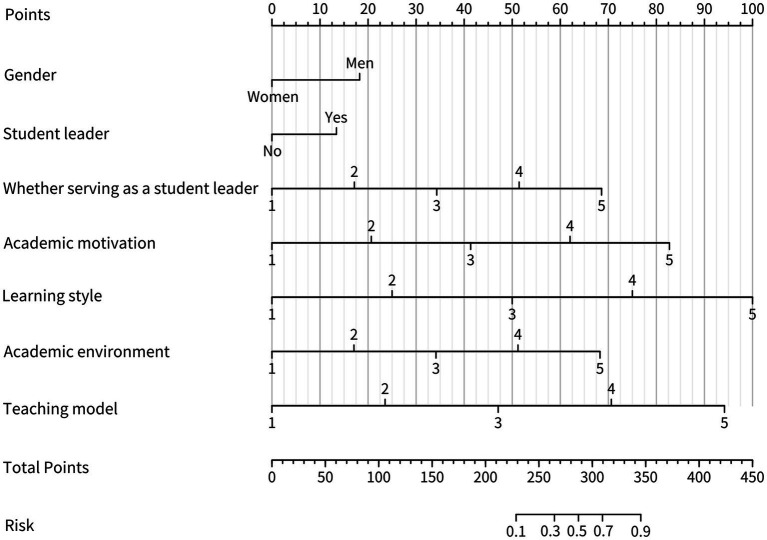
A nomogram predicting the academic innovation capacity of graduate students.

Based on the results of logistic regression analyses, we further constructed a nomogram by combining variables including sex, whether serving as a student leader, teacher-student relationship, academic motivation, learning style, academic environment, and teaching mode. As shown in Figure 2, the values of each factor correspond to the individual scores in the first row of the nomogram; the scores of the seven risk factors are added together to obtain the total score. The higher the total score, the more likely the student is to have a high level of academic innovation. As shown in Figure 2, the values of each factor correspond to the individual scores in the first row of the nomogram; the scores of the seven risk factors are added together to obtain the total score. The higher the total score, the more likely the student is to have a high level of academic innovation. For example, if a female student is a student leader and her self-rating scores for academic motivation, learning style, academic environment, and teaching mode are 4, 5, 5, 5, and 3, respectively, then the nomogram corresponds to 0, 13, 52, 82, 100, 68, and 48, respectively, with a total score of 363, indicating that the probability of the graduate student’s high level of academic innovation is greater than 90%. A high score on the nomogram indicates that the student’s academic innovation ability will be high. Conversely, a low score indicates that the student’s academic innovation ability will be low. Therefore, educators can improve the nomogram score by reinforcing one or more of the influencing factors for different individual students to improve students’ academic innovation ability. Furthermore, educators can also evaluate students’ academic innovation ability potential based on nomogram scores to carry out accurate and efficient training.

**Figure 3:**
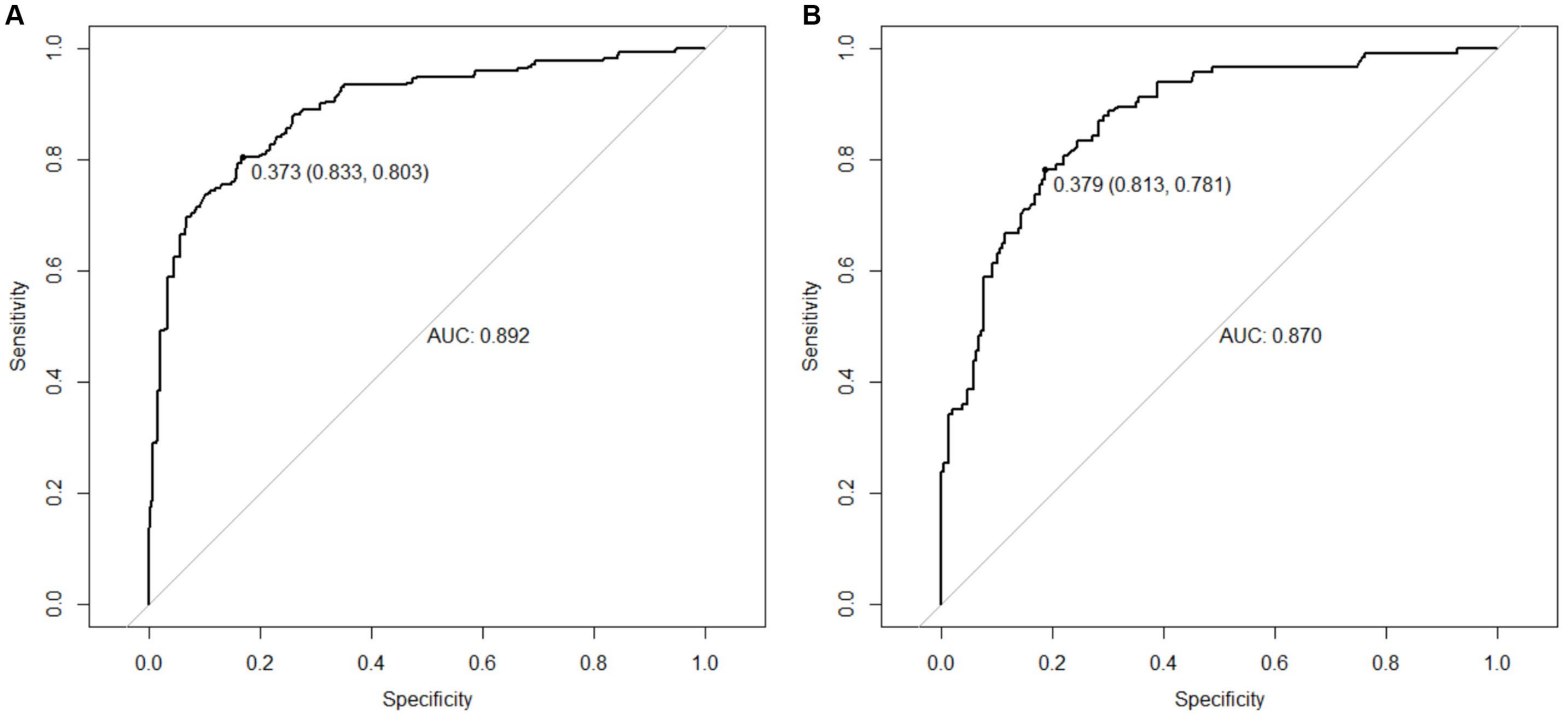
ROC curve of the nomogram prediction model. **(A)** ROC curve of the training set. **(B)** ROC curve of the validation set.

**Figure 4:**
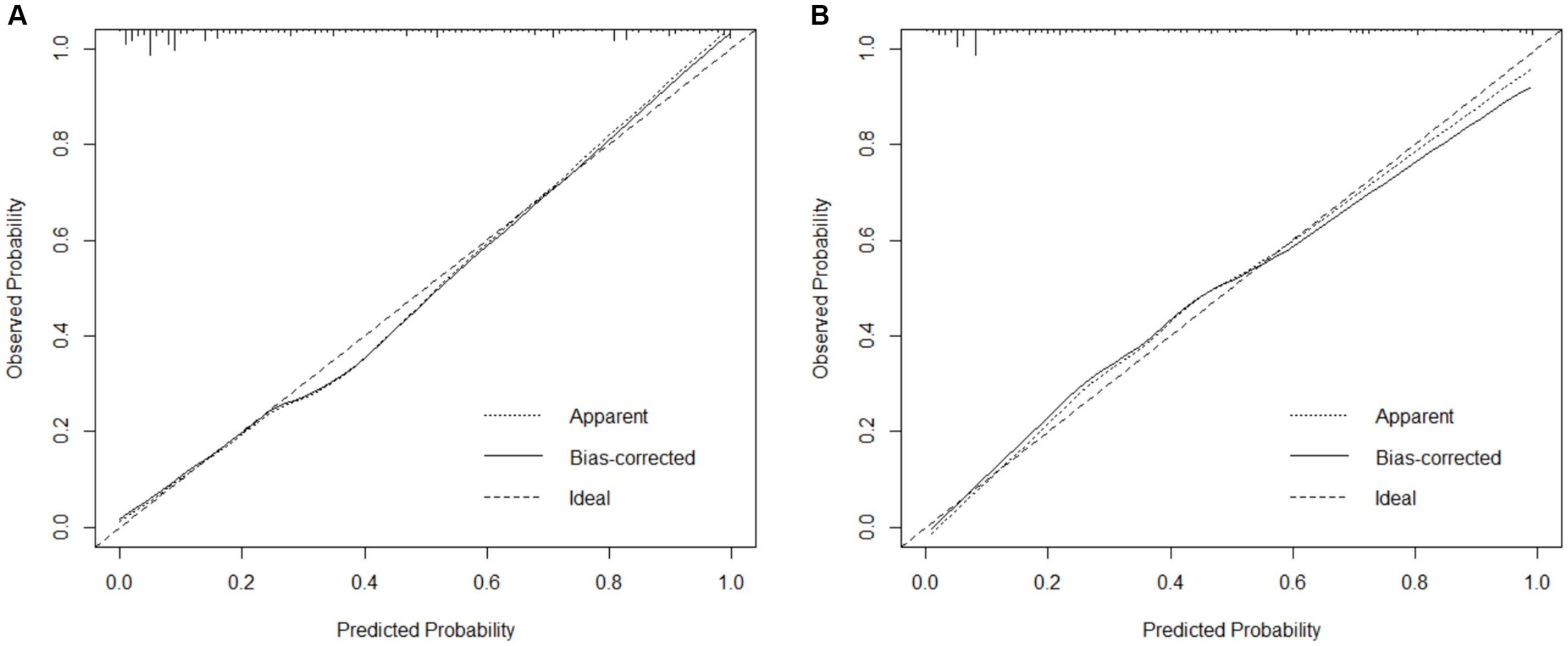
Calibration curves of nomograms. **(A)** Calibration curves of the training set. **(B)** Calibration curves for the test set.

The AUC = 0.892 (95%CI = 0.803 ~ 0.833) of the model indicates that the nomogram has a good degree of discrimination. The model was applied to the test set, as shown in Figure 3, AUC = 0.870 (95%CI = 0.781 ~ 0.813), indicating that the nomogram still had a good degree of discrimination. As shown in Figure 4, the calibration plot shows that the predicted values are in good agreement with the actual values.

## 4 Discussion

In recent years, the impact of the COVID-19 pandemic and various public crises indicates that we should pay attention to public health disciplines and reform the traditional teaching mode according to current times and student characteristics (32). Focusing on the academic innovation ability of graduate students majoring in public health, this study designed a questionnaire to carry out a cross-sectional survey to clarify the current situation and existing problems of graduate students’ academic innovation ability and used logistic regression to determine the influencing factors and predictors of the academic innovation level of graduate students in public health. It provides strong theoretical support for improving the innovation ability of public health graduate students and cultivating more high-quality public health manpower.

The study found that the level of academic innovation of public health graduate students was not high, with an average score of 3.50 ± 0.71, consistent with earlier studies (33). Among them, the professional quality score was the highest, with an average score of 3.65 ± 0.73. This suggests that public health graduate students have a strong sense of career planning and teamwork, which is consistent with other findings (34). In addition, the personal trait score was the lowest (3.37 ± 0.78), especially in the psychological adjustment index (3.29 ± 0.89), which was also consistent with the results of earlier studies (35). Studies have shown that graduate students face heavy research tasks, work uncertainty, interpersonal communication, and other pressures (36, 37), and their self-regulation ability is limited. When students’ psychological pressure is overloaded and their regulation is out of control, negative emotions such as depression and anxiety occur, which affect students’ research progress, career development, academic efficiency, and personal life (38). Therefore, the managers of graduate education should pay attention to strengthening the investment of resources to ensure graduate students’ mental health and the construction of facilities for psychological counseling services to continuously improve their psychological resilience and adaptability.

Personal factors are the foundation of and an important motivation for graduate students’ academic innovation. Among them, academic motivation is closely related to innovation tendency (39), and motivation can be regarded as a necessary “starting ingredient” in the innovation process. In this study, there was a positive correlation between learning motivation and academic innovation ability, and strong learning motivation could promote students’ academic creation. This may be because when students have strong individual initiative, they are more willing to devote energy to tackling challenging tasks, which is consistent with other studies (40, 41) and further validates the role of individual internal factors on academic innovation ability in theory. The learning style of this study emphasizes whether individuals can acquire knowledge stably, continuously, and effectively, and fully discover and make use of surrounding resources. Through the reorganization of existing knowledge and the independent transformation of university resources, students can cultivate independent thinking, problem analysis, and problem-solving skills, which are conducive to creative problem-solving and improving the level of academic innovation (12, 42).

Studies have shown that men have a higher level of academic innovation than women, which is consistent with the results of other studies (43). This may be due to personality differences between genders, resulting in differences in academic innovation. In general, men are more adventurous and independent, which can help inspire innovation. Comparatively, women may be more secure and detail-oriented. This difference may also be related to the way the scale is self-rated (44); men have higher levels of self-esteem than women (45) and men may rate themselves higher when self-rating indicators, leading to a higher level of innovation. In actual research, the diversity of gender composition in the team can promote scientific discovery and innovation potential, so it is necessary to give full play to the creative advantages of different genders (46, 47). Graduate students who serve as student leaders have a higher level of academic innovation ability. During their tenure as student leaders, students improve their planning, cooperation, communication, and time management skills through the completion of organizational activities. These enhancements enable them to integrate resources more effectively, thereby demonstrating greater innovation and practice in academic research (48, 49). Further, they usually have more opportunities and access to a variety of academic resources, which can help them broaden their horizons and up-do-date with the latest research developments, thereby promoting academic innovation.

Moreover, the conclusion that environmental factors affect the academic innovation of graduate students in an indirect way has been widely recognized by scholars. The environmental factors in this study mainly include the academic environment, teacher-student relationship, and teaching mode. Studies have shown that a good academic environment can improve graduate students’ academic innovation ability. The academic environment is the creation of a positive or negative human environment that affects an individual’s ability to innovate academically (50). This study focuses on the attitudes that individuals perceive as being supported or resisted by society and those around them toward academic innovation. By creating a positive environment, students’ intrinsic motivation for scientific research can be stimulated and their enthusiasm for academic innovation can be enhanced (51, 52). In graduate education, the supervisor is primarily responsible for the training of graduate students, and the teacher-student relationship has an important impact on the cultivation of students’ academic innovation ability. A good teacher-student relationship is convenient for full communication and trust between teachers and students. Additionally, tutors can better guide and help students carry out various learning activities, and students also actively absorb experience and knowledge to improve their academic innovation ability; similar results have been found in other studies (16, 53, 54). Teaching mode is also an important factor affecting graduate students’ academic innovation ability. Encouraging academic innovation through teaching mode can improve students’ sense of innovation, resulting in creativity and students actively carrying out scientific research. Research shows that the multidisciplinary integrated teaching mode can promote the cultivation of students’ academic innovation ability. By integrating other disciplines into public health teaching, students can broaden their horizons and cultivate interdisciplinary talents, using new knowledge and skills in multiple fields to create academics and improve their academic innovation ability (15, 55, 56). Some scholars have proposed the collaboration-inquiry teaching model, which is organized and implemented in three rounds, proving that this teaching model can cultivate and improve students’ innovation ability (57). By reforming the traditional teaching model, students can be stimulated to independently innovate (58). In this study, a good academic environment, teacher-student relationship, and teaching mode all positively affect academic innovation ability and confirm the importance of implementing these environments, relationships, and modes to improve graduate students’ academic innovation ability of in public health.

The nomogram constructed in this study can be used to evaluate the academic innovation level of public health graduate students in China. The AUC = 0.892 (95%CI = 0.803 ~ 0.833) of the nomogram indicated that the nomogram had good discrimination, and the calibration chart showed that the predicted value was in good agreement with the actual value. Based on the above findings, the training institutions of public health graduate students can rely on this nomogram to quickly identify students whose academic innovation ability needs to be improved and take targeted measures, such as enhancing learning motivation, improving teaching mode, and optimizing the academic atmosphere. This effectively responds to the challenges of public health education, helps universities to cultivate more high-quality talents with both innovation and practice, and provides a scientific basis and practical path for the high-quality innovation and development of public health graduate education in China.

There are some limitations in this study. First, the survey subjects are mainly from one region of China, and there may be an issue of regional homogeneity of the sample. Thus, in future research, it is necessary to further expand the sample size, carry out the survey in more regions of China, and conduct inter-regional comparative analysis. Second, we mainly used self-reported measurements from respondents, which were prone to bias. This may be further solved by adding categories of survey respondents. For example, the survey data from the perspective of graduate students’ tutors can be collected at the same time; the results of other people’s evaluations can be compared with the students’ self-evaluation and the results of the comparative analysis can be supplemented by the results of this study.

## 5 Conclusion

The influencing factors of public health graduate students’ academic innovation ability mainly include gender, whether they are currently serving as student leaders, teacher-student relationship, academic motivation, learning style, academic environment, and teaching mode. The nomogram model constructed in this study can be used to predict the academic innovation level of public health graduate students, which is helpful for university education administrators to evaluate students’ academic innovation ability based on nomogram scores and carry out accurate and efficient training.

## Data Availability

The raw data supporting the conclusions of this article will be made available by the authors, without undue reservation.
